# Evaluation of the wound healing efficacy of new antibacterial polymeric nanofiber based on polyethylene oxide coated with copper nanoparticles and defensin peptide: An *in-vitro* to *in-vivo* assessment

**DOI:** 10.1016/j.heliyon.2024.e29542

**Published:** 2024-04-10

**Authors:** Sahba Eslaminezhad, Farhad Moradi, Mahmoud Reza Hojjati

**Affiliations:** a Sahba Eslaminezhad, Department of chemical engineering, Shiraz Branch, Islamic Azad University, Shiraz, Iran; b Farhad Moradi, Department of Bacteriology & Virology, School of medicine, Shiraz University of Medical Sciences, Shiraz, Iran; c Mahmoud Reza Hojjati, Faculty of Engineering, Department of Chemical Engineering, Shiraz Branch, Islamic Azad University, Shiraz, Iran

**Keywords:** Electrospinning, Nanocomposite, Wound healing, Polyethylene oxide, Defensin, Copper nanoparticles

## Abstract

**Objective:**

Today, designing nanofibers with antibacterial properties using electrospinning technology is one of the attractive approaches for wound healing.

**Methods:**

*& analysis*: This study aims to fabricate a nanocomposite from polyethylene oxide (PEO) coated with copper nanoparticles (NPs) and defensin peptide with wound healing and antimicrobial properties in different ratios of CuNPs/defensin (2/0 mg), (1.5/0.5 mg), and (1/1 mg) in the fixed contain polymer (98 mg). Then, the nanofiber properties were investigated by SEM, tensile, DSC, and BET analysis. Also, the antibacterial properties against *S. aureus* and *E. coli*, antioxidant, and *in-vivo* wound healing effects and histological analysis of the designed nanocomposites were evaluated in rat models.

**Results:**

Our SEM images showed that CuNPs and defensin were properly coated on the PEO surface. According to the tensile, DSC, and antibacterial analysis results, the most appropriate feature was related to CuNPs/defensin (1.5/0.5 mg), with maximum elasticity, heat resistance, and antibacterial activity. Furthermore, the designed nanocomposites showed the best performance as a wound closure agent by increasing dermis and epidermis volume density, stimulating fibroblast cells and collagen fiber production, and improving skin vessels.

**Conclusion:**

According to our results, PEO nanofibers loaded with CuNPs and defensin have the best potential for wound healing, and they can be used as antibacterial materials in the textile, drug, and medical industries.

## 1 Introduction

Wounds are an important global healthcare issue that has been defined as a "silent epidemic" due to their prevalence and profound effects on global health. Overall, it has been estimated that chronic wounds alone affect 1–2% of the population in developed countries. Because the occurrence of chronic wounds is related to age and other age-related diseases such as diabetes, it can become a big problem. In developed countries, chronic wound care costs account for 1–6% of total health care costs. In general, chronic wounds have the most adverse effects on people over 65 years old. According to reports, due to the increase in the elderly population in the United States, chronic wounds will become a very serious problem [1]. The care of chronic wounds and the use of antimicrobial dressings have many effects on the economic system and global healthcare markets. Additionally, statistics and analysis will show an increase in the annual growth rate of billions of dollars in different countries, including the United States, China, Japan, Asia, Oceania, Australia, India, South Korea, and Canada, in the period 2020–2027 [1,2]. Chronic wounds become challenging when they are infected with microbial agents. As public health awareness of the effects of pathogens on personal hygiene and health risks has increased in recent years, many researchers have tried to minimize microbial infections. Wound infection and the spread of infection through hospital textiles are serious concerns in hospitals. Therefore, several studies have been conducted to produce antimicrobial textiles in the past few years [[3], [4], [5]]. The main challenge of this research is the requirement for materials that do not cause toxicity, allergies, or sensitivities. In addition, the emergence of multidrug-resistant bacteria is very challenging [6]. Therefore, the development of new and effective antimicrobial treatments is still an important research issue. Over the past few years, new antibacterial materials have been developed, including Quaternary Ammonium Compounds, metal nanoparticles, and plant antimicrobial peptides [[7], [8], [9]]. As a promising alternative, plant peptides are a new group of antimicrobial agents with a low propensity to induce drug resistance. Plant peptides, extracted from plants such as soy, are small, cationic, and amphiphilic peptides. These peptides are part of the innate immunity and exhibit broad-spectrum antimicrobial activity. The mechanism of interaction of plant antimicrobial peptides with microbes involves the penetration of peptides into cells, the disruption of lipid membranes, increased membrane permeability, and rapid cell death. Defensin is one of the most effective antimicrobial peptides produced by the innate immune system of plants. These small and cationic peptides have eight cysteines in their molecules to create a strong structure by forming four disulfide bonds, which allows them to have maximum activity against the membranes of microbes [[10], [11], [12]]. Furthermore, various types of metal-based nanoparticles with antimicrobial properties, such as silver, zinc, gold, copper, and magnesium, have been developed. Compared to other metallic nanoparticles used in antimicrobial applications, biocompatible Cu nanoparticles (NPs) exhibit several advantages, including lower cost and easy production. Cu NPs interact with the bacterial cell wall and cause cellular damage. In fact, Cu NPs can induce oxidative stress in cells by generating reactive oxygen species that damage proteins, lipids, and nucleic acids [12,13]. The use of plant peptides along with antibacterial nanoparticles has the potential to increase the effectiveness of antimicrobial properties. Nowadays, one of the best methods for designing nanofibers or wound dressings containing different antimicrobial compounds is the use of the electrospinning technique. Nanofiber membranes prepared by electrospinning have excellent properties, such as a high specific surface area and strong plasticity, allowing for the manipulation of nanofiber components for intended purposes in biomedical applications. In the last two decades, with rapid progress in understanding the importance of electrospinning, the applications of electrospun nanofibers have expanded, and nanofibers have been introduced with a wide range of applications in various fields, including medicine, filtration, and textiles [[14], [15], [16]]. The electrospun nanofibers incorporated with antimicrobial agents have been fabricated for antimicrobial applications [17,18]. Moreover, electrospinning is a simple, affordable, and available technology for the preparation of polymer nanofibers, which has attracted extensive scientific and industrial attention in the past few decades due to its versatility and ability to fabricate nanofiber networks with high adjustability. With the increasing popularity of nanomaterials in the current century, electrospun nanofibrous membranes are used in various fields of medicine, pharmacy, dentistry, and tissue engineering. Today, polymer-based nanocomposites have received much attention for designing dressings. These nanocomposites contain multi-element materials or at least one element at the nanoscale, which have different properties. There are various polymers that can be used to make nanocomposites, including polyethylene oxide, polyvinyl alcohol, polyglycolic acid, cellulose acetate, chitosan, collagen, polybutylene succinate, and alginate. Since PEO has attractive properties that facilitate electrospinning, it is used as a basic material in nanocomposite synthesis in this study. Biocompatibility, flexibility, high water solubility, narrow or broad molecular weight distribution, high surface area, high crystallinity, and non-toxicity are among the characteristics of this polymer [[18], [19], [20], [21], [22]]. Considering the importance of the wound and its destructive effects, as well as the scientific advances in the field of producing nanofibers using the electrospinning technique, designing new nanofibers with compounds that have antibacterial properties is very important. Due to the significance of this issue, in this research, we attempted to fabricate a new polymer-based nanocomposite using the electrospinning method. Therefore, our goal in this research was to synthesize a nanocomposite with antimicrobial properties based on PEO/CuNPs/defensin using the electrospinning method. To optimize the nanocomposite for high performance, different ratios of antimicrobial agents, Cu NPs, and defensin, in the fixed polymer phase (98%), were prepared. Finally, the efficacy of this nanocomposite in wound healing was investigated.

## 2 Materials and methods

### 2.1 Materials

To design the polymer-based nanocomposite, polyethylene oxide (MW = 44.02 g/mol) and defensin peptide (purity ≥95% and MW = 3.5 kDa) were purchased from Sigma Aldrich. Additionally, copper nanoparticles (MW = 76.54 g/mol, 20–40 nm) were obtained from US NANO, and acetic acid 96% was purchased from Merck Millipore. To evaluate the antibacterial efficacy of the designed nanocomposite against gram-negative and gram-positive bacteria, we used *Staphylococcus aureus* ATCC 23235 and *Escherichia coli* ATCC 25922 standard strains (−80 °C stock, Department of Bacteriology, Shiraz University of Medical Sciences).

### 2.2 Experimental preparation of the nanocomposite

To prepare the nanocomposite, first, 0.3 ml of copper nanoparticles was dissolved in 100 ml of 50% acetic acid using a stirrer at 50 °C. Then, 0.3 ml of defensin peptide was dissolved in 100 ml of 1 M acetic acid at 40 °C. Furthermore, 5 g of polyethylene oxide were combined with 15 ml of deionized water at 60 °C. Finally, these prepared solutions were used to create nanocomposites with different ratios of defensin to copper nanoparticles. For this purpose, 2 ml of the copper nanoparticle solution was added dropwise to 98 ml of polyethylene oxide and mixed at 50 °C (Cu NPs/defensin 2/0). The second sample was prepared by adding 1 ml of defensin peptide and 1 ml of copper nanoparticles to 98 ml of polyethylene oxide (Cu NPs/defensin 1/1). The third sample was obtained by adding 1.5 ml of copper nanoparticles and 0.5 ml of defensin peptide to 98 ml of polyethylene oxide (Cu NPs/defensin 1.5/0.5). Finally, the pure polyethylene oxide solution was considered as the negative control sample. After preparing the samples, they were used for the electrospinning process**.**

### 2.3 Electro spinning and nanofibers preparation

Electrospinning and nanofiber preparation were accomplished according to Yu L et al., with some modifications [23]. Nanofiber preparation was performed in the Central Research Laboratory of Shiraz University of Medical Sciences using an electrospinning instrument (ES2000, Nano Fanavaran Meghyas Iran). In this process, each solution from the previous stage was aspirated with a 10 ml syringe and sprayed onto aluminum sheets placed on the collector drum. The power supply was set to a voltage of 21 kV, and the flow rate of the solution was determined by setting up the syringe pump at 1 ml/h. The rotational speed of the collector drum, based on preliminary tests, was 2500 rpm, and its distance to the nozzle was set to 10 cm. During the experiments, relative humidity and temperature values ranged from 35 to 42% RH and 25–35 °C, respectively. Finally, uniform nanofibers with dimensions of 25 × 30 square meters were provided for further analysis.

### 2.4 Nanocomposite characterization

#### 2.4.1 Morphology and particle size

In this study, the morphology and particle size of the nanocomposites were recorded using a scanning electron microscope (SEM) (HITACHI S-4160 model, Vacc = 25 kV). The samples were coated with a thin layer of gold and analyzed with Image J software.

#### 2.4.2 Tensile analysis of nanocomposites

The mechanical properties and tensile rate of nanocomposites were investigated through a tensile test. In this analysis, our samples were prepared in sizes of 0.5 × 2.5 cm and placed inside the tensile test machine (universal testing machine (UTM)). Furthermore, in this stage, we used ASTM D638 and ISO 527 as standards, and the samples were stretched at a speed ranging from 0.2 to 20 mm/min.

#### 2.4.3 DSC analysis of nanocomposites

Thermal analysis, which measures the energy value of the exothermic or endothermic reaction, was performed on the nanocomposites using Differential Scanning Calorimetry (DSC) with TA 2010 Instruments in New Castle. Prior to the DSC analysis, the nanocomposites (approximately 5 mg) were dried at 70 °C for 4 h, and all measurements were conducted in a nitrogen atmosphere. The endothermic behavior of the designed nanocomposites during the melting process was finally analyzed [24].

#### 2.4.4 Minimum inhibitory/bactericidal concentration (MIC/MBC)

In order to investigate the antibacterial effects of the designed nanocomposite, we performed the microbroth dilution method in 96-well microplates according to the CLSI 2021 guideline [25]. For this purpose, *S. aureus* ATCC 25923 and *E. coli* ATCC 25922 were prepared to determine the minimum inhibitory concentration (MIC) and the minimum bactericidal concentration (MBC). In our study, a two-fold serial dilution of Solution A (PEO/Cu NP (98/2 mg)), Solution B (PEO/Cu NPs/Defensin (98/1.5/0.5 mg)), Solution C (PEO/Cu NPs/Defensin (98/1/1 mg), and Solution D (PEO 100 mg) was prepared in the nutrient broth medium (100 μg/ml per well). In addition, the bacterial suspension was adjusted to 1.5 × 10^8^ CFU/ml and 100 μg/ml of it was added to each well. Finally, the microplate was incubated at 37 °C for 24 h. The results were evaluated based on the growth or non-growth of the standard strains in the presence of different concentrations of the nanocomposite. In order to ensure the determination of MIC and MBC concentrations, the contents of the wells were extracted, cultured in separate nutrient agar media, and incubated at 37 °C for 24 h.

#### 2.4.5 BET analysis of nanocomposite

The Brunauer-Emmett-Teller (BET) analysis is used to measure the specific surface area and density of nanomaterials. Among the methods used to determine porosity, the BET method, which is based on absorption, has received attention. First, the samples were placed at 120 °C for 2 h. Then, the samples were placed in the liquid nitrogen tank, and the amount of gas absorbed and desorbed by the material was calculated by gradually increasing and decreasing the nitrogen gas pressure in each step. Finally, the graph of the volume of nitrogen gas absorbed and desorbed by the material at a constant temperature was drawn.

### 2.5 Antioxidant test

In the present study, the antioxidant effect of the designed nanofibers was investigated by preparing a methanolic solution of 2, 2-diphenyl-1-picrylhydrazyl (DPPH) with a concentration of 0.110 mM. Then, 180 μl of the prepared methanolic DPPH solution was mixed with 20 μl of different concentrations of PEO/Cu Nps/defensin (100/0/0, 98/2/0, 98/1/1, 98/1.5/0.5 mg/ml) in a 96-well plate and incubated for 30 min at 25 °C in the dark. Finally, absorbance measurements were carried out at a wavelength of 517 nm [26].

### 2.6 *In-vivo* and histological analysis

This experiment was conducted based on the guidelines of the National Institute of Health (NIH) and confirmed by the Animal Care Committee of Shiraz University of Medical Sciences (IR.SUMS.REC ethical code: 16340201972002). In this study, animal models were used to investigate the performance of the designed nanofibers in wound healing. Twenty healthy male Wistar rats (aged 8–10 weeks with a weight of 250–300 g and a mass index over 30 g/cm2) were obtained from the Shiraz University of Medical Sciences animal house. The rats were housed in separate polycarbonate cages at 26–28 °C, 85% humidity, and a 12-h dark cycle with standard water and food. After anaesthetising the rats with an intramuscular injection of xylazine (15 mg per kg body weight) and ketamine (60 mg per kg body weight), the hair on the backs of the rats was shaved using an electric shaver. Then, to create second-degree burns, metal stamps with dimensions of 1*1 cm, which had been placed in boiling water at 90 °C for 3 min, were applied to the backs of the rats for 5 s. Then, the rats were randomly divided into five groups. The first group was considered a negative control. Furthermore, the second to fifth groups of rats were daily treated with polyethylene oxide/copper (98/2 mg), polyethylene oxide (100 mg), polyethylene oxide/copper/defensin (98/0.5/1.5 mg), and polyethylene oxide/copper/defensin (98/1/1 mg) respectively. Then, the healing of the wounds created on the first, fourth, seventh, and fourteenth days was evaluated by measuring the wound diameter. After fourteen days, when the wounds of some groups were closed, a histological examination was conducted by anaesthetising rats with intramuscular injections of xylazine (15 mg per kg of body weight) and ketamine (60 mg per kg of body weight). For histological analysis, we prepared 1*1 cm biopsy specimens and transported them to our laboratory in 10% neutral buffered formalin as a fixative solution. The specimens were placed in the formalin fixative for 24 h prior to histological processing. Then, paraffin blocks were made from the specimens and thin microscopic sections (3–5 μm) were prepared. In this study, we used Masson's trichrome staining to evaluate our paraffin-embedded, formalin-fixed tissues according to Nathan Chandler Foot (1933) [27]. In summary, for this staining, the specimens were first deparaffinized and hydrated with distilled water, and then stained with various solutions including Weigert's iron hematoxylin, Biebrich scarlet-acid fuchsin, phosphotungstic/phosphomolybdic acid solution, and aniline blue, following the standard protocol. After staining, collagen fibers (blue), muscle fibers (red), and cell nuclei (black) can be differentiated under light microscopic evaluation. Additionally, the average surface area of the wound, the cell volume of the epidermis and dermis, the number of fibroblast cells, the volume density (Vv (collagen/dermis)) of collagen fibers, vessel density, and longitudinal measurements of all groups were studied using image analysis software. To determine the wound healing rate, post-hoc parametric LSD test and ANOVA were used.

## 3 Results

### 3.1 SEM analysis results

**Fig. 1:**
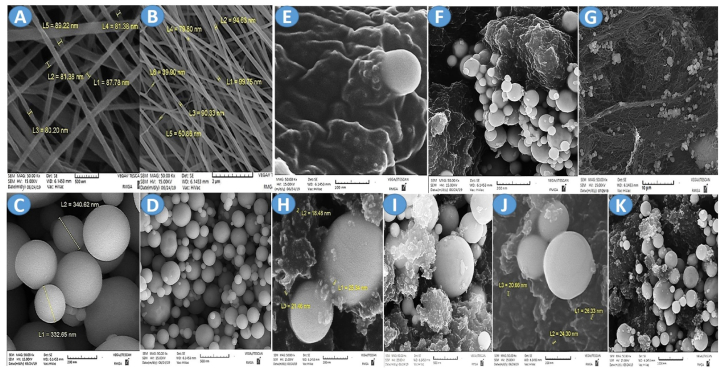
The SEM images of PEO nanofiber (A) scale bar 500 nm, (B) scale bar 2 μm; Cu NPs (C) scale bar 200 nm, (D) scale bar 500 nm; PEO/Cu NPs (98/2 mg) (E) scale bar 100 nm, (F) scale bar 200 nm, (G) scale bar 10 μm; PEO/Cu NPs/Defensin (98/1/1 mg) (H) scale bar 200 nm, (I) scale bar 500 nm; PEO/Cu NPs/Defensin (98/1.5/0.5) (J) scale bar 200 nm, (K) scale bar 1000 nm.

Fig. 1A and B shows the SEM images of polyethylene oxide nanopolymer obtained by electrospinning in two scales of 2 μm and 500 nm. Fig. 1 A clearly shows pure polyethylene oxide nanofibers on a scale of 500 and confirms the nanosize of the fibers. Moreover, the diameter of the particles is between 30 and 99 nm. According to Fig. 1B, polyethylene oxide polymer nanofibers have been correctly synthesized. One of the features of this synthesis is the linear placement of the nano strands, parallel to each other, without overlapping, and the absence of intersection. Additionally, the scaling of nanoparticles is in the range between 30 and 90 nm, which indicates that the sample is a nanopolymer. In Fig. 1C and D, copper nanoparticles are shown in two scales of 500 nm and 200 nm, with a spherical morphology, no aggregation, a size of 90 nm, and high purity. Furthermore, in Fig. 1E, F, and 1G, polyethylene oxide nanopolymer and copper nanoparticles are shown in three scales of 100 nm, 200 nm, and 10 μm. The copper nanoparticles are embedded in the surface of polyethylene oxide nano strands while maintaining their spherical morphology. Fig. 1H and I shows the formation of polyethylene oxide nanopolymer/copper nanoparticles/peptide (98/1/1 mg) and the adhesion of copper nanoparticles and peptide on the surface of polyethylene oxide strands without creating clumps. Additionally, Fig. 1J and K shows the formation of polyethylene oxide nanopolymer/copper nanoparticles/peptide (0.5/1.98 mg), with a higher amount of copper compared to the peptide. The copper nanoparticles and peptide are properly placed together, and the nanofibers are properly coated. The size of the peptide particles is between 17 and 25 nm.

### 3.2 Tensile analysis

**Fig. 2:**
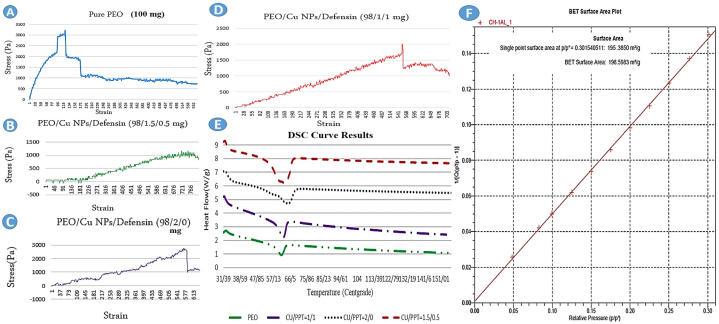
(A) Tensile stress-strain curve of pure PEO nanofiber, (B) Tensile stress-strain curve of PEO/CuNPs/defensin (98/1.5/0.5 mg) nanocomposite, (C) Tensile stress-strain curve of PEO/CuNPs/defensin (98/2/0 mg) nanocomposite, (D) Tensile stress-strain curve of PEO/CuNPs/defensin (98/1/1 mg) nanocomposite. (E) DSC analysis results of nanocomposites (PPT: Peptide). (F) BET curve of PEO/CuNPs/defensin (98/1.5/0.5 mg) nanocomposite.

The mechanical properties of the designed nanocomposites were evaluated using a tensile test, and the related stress-strain curves were designed based on ASTM D 882 (Fig. 2A–D). The tensile strength of a material accounts for how much stress the material will bear before suffering permanent deformation or tearing. According to the tensile strength diagrams, the highest tensile strength and elasticity are related to PEO/Cu NPs/defensin (98/1.5/0.5) and PEO/CuNPs/defensin (98/1/1 mg) nanocomposites, respectively. On the contrary, the lowest resistance was observed in PEO nanofibers. The results showed that the highest elongation at break (maximum tensile strain and the highest flexibility on the horizontal axis) was obtained for the PEO/Cu NPs/defensin (98/1.5/0.5) nanocomposite. On the contrary, the least elongation at break belongs to pure polyethylene oxide (PEO). Besides, according to the results, the addition of nano copper has increased the tensile strength and mechanical properties of the composite. For example, in the case of the nanocomposite containing nano copper, it has a higher tensile strength than pure PEO. The increase in tensile strength is probably related to the amount of nanoparticles, fine dispersion between polymer chains, and the interaction of the surface charge of nano copper with the hydroxyl group of defensin and the structure of PEO. In general, the analysis of the graphs shows that the fibers will be more resistant with the addition of nanoparticles.

### 3.3 DSC analysis results of nanocomposites

Differential scanning calorimetry (DSC) is an analytical thermal method used to study the thermal properties of polymers. The DSC diagram is plotted based on heat flux versus temperature and is performed in a dry nitrogen atmosphere in the temperature range of 151-31 °C at a rate of 10 °C/min. According to Fig. 2E, nanofibers containing additive compounds have the highest thermal stability compared to pure nanofibers due to the presence of hydrogen bonds between molecules and electrostatic interactions between functional groups of PEO, nano copper, and defensing peptide. The DSC curve shows the endothermic behavior of PEO, CuNPs, and defensin during the melting process (Fig. 2E). It is also observed that the melting reserve of PEO nanofibers, as a control, is close to the melting temperature of PEO/CuNPs/defensin. Moreover, by increasing the amount of Cu NPs, the melting point of the nanocomposite increases. PEO/Cu NPs/defensin (98/1.5/0.5) and PEO nanofibers show the highest and lowest thermal resistance, respectively.

### 3.4 The result of BET analysis

BET analysis is used to measure the specific surface area and porosity level of nanofibers. This test is performed by measuring the volume of nitrogen gas absorbed and desorbed by the surface of the nanofibers at a fixed temperature of liquid nitrogen (77 K) and a relative pressure of about 0.3–0. In this study, the BET test was conducted on the selected nanocomposite with the best antimicrobial effect, PEO/Cu NPs/defensin (98/1.5/0.5 mg). The results were presented in the form of a linear graph, and the specific surface area of the nanofibers was determined to be 198.5983 m2/g (Fig. 2F).

### 3.5 Antibacterial activity

**Fig. 3:**
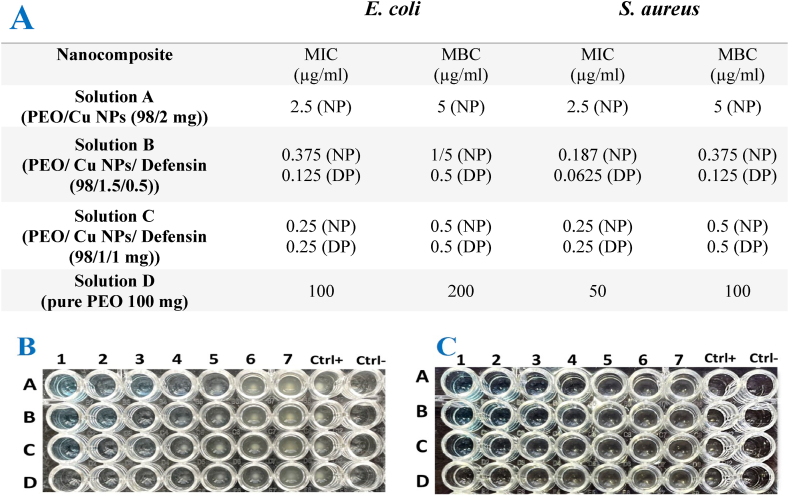
Antibacterial susceptibility test Results. Nanoparticles (NP), Defensin Peptide (DP). (A); Table show Minimum Inhibitory/Bactericidal Concentrations (MIC & MBC) for solution A-D. (B) and (C): 96 microplate results for serial dilution of Solution A (A1-A7 = PEO/20–0.312 Cu NPs μg/ml), Solution B (B1–B7 = PEO/1.5–0.023 Cu NP/0.5–0.0078 Defensin μg/ml), Solution C (C1–C7 = PEO/1–0.0156 Cu NPs/1–0.0156 Defensin μg/ml), Solution D (D1-D2 = 1–0.0156 μg/ml; pure PEO) against *E. coli* and *S. aureus.*

Our results showed the best antibacterial activity of the designed nanocomposite against gram-positive and gram-negative standard strains. Although solution D, pure PEO; 1–0.0156 μg/ml, has antibacterial activity, its MIC can be decreased after loading with Cu NP and defensin peptide. These results present the best antibacterial efficacy of the PEO 98/1.5–0.023 CuNP/0.5 0.0078 Defensin μg/ml solutions. More information about MIC & MBC of all nanocomposite were compared with more details in Fig. 3A. Besides, the result of serial dilutions of all nanocomposite in 96 microplate against *E. coli* and *S. aureus* were characterized in Fig. 3B and C.

### 3.6 Antioxidant results

Based on the antioxidant results, the highest antioxidant activity is associated with PEO/Cu NPs/defensin nanofiber at concentrations of 98/1.5/0.5, 98/1/1, and 98/2/0 mg/ml, displaying DPPH radical scavenging abilities of approximately 23.5, 20, and 16.2, respectively. Lastly, the sample that solely consisted of PEO polymer lacks any antioxidant properties. This evidence demonstrates that defensin is a natural agent with antioxidant properties, capable of eliminating free radicals.

### 3.7 Wound healing assay

#### 3.7.1 Wound closure assay

**Fig. 4:**
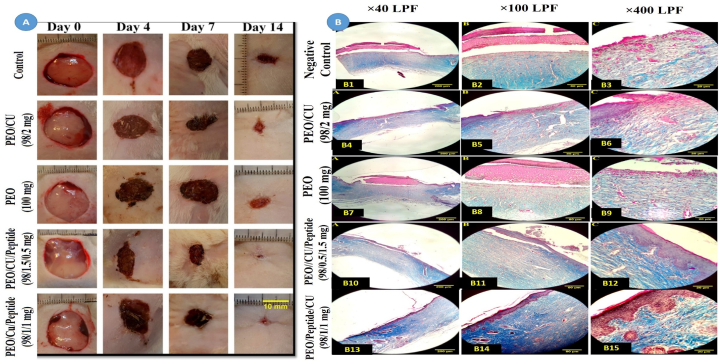
(A); Macroscopic picture of the wound healing process in different groups on days 0, 4, 7, and 14. (B); Masson's trichrome staining and light photomicrograph of the wound site in different groups on the 14th day; B1–B3: negative control group, B4–B6: PEO/Copper (98/2 mg) receiving group, B7–B9: PEO (100 mg) receiving group, B10–B12: PEO/Copper/Peptide (98/0.5/1.5 mg) receiving group, B13–B15: PEO/Copper/Peptide (98/1/1 mg) recipient group.

The wound healing process was investigated by measuring and comparing the average wound area in different groups on the 0th, 4th, 7th, and 14th days, as shown in Fig. 4A and Table 1. A significant difference between the negative control, polyethylene oxide (PEO 100 mg), PEO/copper (PEO/Cu (98/2 mg)), PEO/copper/peptide (PEO/Cu/peptide (98/1/1 mg)), and the PEO/copper/peptide (PEO/Cu/Peptide (98/1.5/1 mg)) groups was analyzed using ANOVA and post hoc LSD parametric test. According to Table 1, there is a significant decrease in the average wound area on the 4th and 14th days in the recipient groups of PEO/Cu/peptide (98/1/1 mg) and PEO/Cu/Peptide (98/1.5/1 mg) compared to the negative control group of PEO (100 mg) and the PEO/Cu (98/2 mg) group. There was no significant difference (p < 0.05) among the other groups on the 7th and 14th days. Further details can be found in Table 1.

**Table 1:** The results of the average wound closure area in the studied groups on days 0, 4, 7, and 14. Data are shown as mean ± SD. (*) Significant difference compared to the control group. (†) Significant difference compared to the group receiving PEO. ($) Significant difference compared to the group receiving PEO/Cu. (*, †, $) Significant differences at the p < 0.05 level. (**, ††, $$) Significant differences at the p < 0.01 level. ($$$) Indicates a significant difference at the p ≤ 0.001 level.

Wound closure area (mm^2^) (Mean ± SD)				
Group	0 Day	4^th^ Day	7^th^ Day	14^th^ Day
Control^-^	212.66 ± 10.68	195.28 ± 14.56	106.06 ± 8.53	23.64 ± 4.08
PEO/CU (98/2 mg)	216.50 ± 12.23	190.10 ± 18.63	115.16 ± 16.56	20.20 ± 1.81
PEO (100 mg)	229.05 ± 9.79	177.71 ± 17.70	119.13 ± 15.66	24.94 ± 4.52
PEO + CU + Peptide (98/1.5/0.5 mg)	226.68 ± 6.82	137.37 ± 12.88*†	64.78 ± 4.15*††$$	9.11 ± 1.89**†$$$
PEO + Cu + Peptide (98/1/1 mg)	230.56 ± 13.89	141.11 ± 11.51*†	55.51 ± 5.16**††$$	8.29 ± 1.65**†$$$

#### 3.7.2 Microscopic and macroscopic tissue examination

Macroscopic evaluation of the wound healing process was conducted in different groups on days 0, 4, 7, and 14 (Fig. 4B). Also, Masson's trichrome staining and light photomicrographs of the wound site in different groups on the 14th day are shown in Fig. 4B. According to the macroscopic results, the PEO/Cu/peptide (98/1/1 mg) and PEO/Cu/Peptide (98/1.5/0.5 mg) recipient groups exhibited better wound healing and wound closure percentages compared to the other groups until the 14th day. Furthermore, Masson's trichrome staining revealed that the negative control group exhibited inflammatory exudate, absence of migration of keratinocytes, hypercellular and low-fibrous granular tissue with visible edema. Neutrophils were observed under the clot and the epithelial tissue was incomplete (Fig. 4 B1–B3). Therefore, the PEO (100 mg) receiving group showed similar conditions to the negative control group (Fig. 4 B4–B6). In the PEO/copper (98/2 mg) receiving group, the granulation tissue was hypercellular, less fibrous, and exhibited edema. The migration of keratinocytes and the prominence of the edge of the epithelial tissue were observed, but the epithelial tissue was still incomplete (Fig. 4 B7–B9). Additionally, in the group receiving PEO/Peptide/copper (98/1/1 mg), the wound space was filled with mature granulation tissue that contained dense and mature collagen strands. A thick layer of squamous tissue was also visible on the surface of the wound (Fig. 4 B10–B12). In contrast, in the group receiving PEO/Peptide/copper (98/0.5/1.5 mg), the wound space was filled with mature granulation tissue that contained dense and mature collagen fibers. Furthermore, there was a significant increase in the number of fibroblasts. Similar to the previous group, a thick coating of squamous tissue could be seen on the surface of the wound (Fig. 4 B13–B15).

#### 3.7.3 Volume density of the epidermis and dermis

Based on the statistical results related to the average volume density of the epidermis in the investigated groups on the 14th day, there was a significant increase in the average volume density of the skin epidermis in the PEO/Cu/peptide (98/1.5/0.5 mg) and (98/1/1 mg) recipient groups compared to the negative control group. The results related to the average volume density of the dermis layer in the examined groups on the 14th day showed that there was no significant difference (p < 0.05) among the examined groups. More detailed information is compared and mentioned in Table 2.

**Table 2:** The results of the average volume density of the epidermis and dermis of the skin in the studied groups on the 14th day. Data are shown as mean ± SD. ** and † indicates a significant increase in the average volume density of the skin epidermis in the receiving group PEO/Cu/peptide (98/1.5/0.5 mg) and (98/1/1 mg) compared to the control group (p = 0.010, p = 0.014), and PEO group (p = 0.008 and p = 0.011), respectively. The results of the volume density of the skin dermis show no significant difference between the studied groups.

Group	A; Volume Densities of the Epidermis (Mean ± SD)	B; Volume Densities of the Dermis (Mean ± SD)
Control	0.160 ± 0.05	0.808 ± 0.06
PEO/CU (98/2 mg)	0.164 ± 0.03	0.835 ± 0.03
PEO (100 mg)	0.212 ± 0.04	0.788 ± 0.04
PEO + CU + Peptide (98/1.5/0.5 mg)	0.240 ± 0.04**†	0.823 ± 0.06
PEO + Cu + Peptide (98/1/1 mg)	0.232 ± 0.03**†	0.829 ± 0.06

#### 3.7.4 Fibroblast cells, collagen fibers, and skin vessels

The average results of the number of fibroblast cells, the volume density of skin collagen fibers, the volume density of skin vessels, and the longitudinal density of vessels in the studied groups on the 14th day are completely compared in Table 3. According to the results, there is a significant increase in the number of fibroblast cells and the average volume density of skin collagen fibers in the recipient PEO/Cu/peptide (98/1.5/0.5 mg) and (98/1/1 mg) groups compared to the negative control and other groups. More detailed information is provided in Table 3.

**Table 3:** The average results of the number of fibroblast cells, volume density of skin collagen fibers, volume density of skin vessels, and longitudinal density of vessels in the studied groups on the 14th day. Columns A: (**) a significant increase in the number of fibroblast cells in recipient groups (PEO/CU/Peptide; 98/1.5/0.5 mg) and (PEO/CU/Peptide; 98/1/1 mg) in compared to the control group at the level of p = 0.005. (††) a significant increase in the number of fibroblast cells in recipient (PEO/CU/Peptide; 98/1.5/0.5 mg) and (PEO/CU/Peptide; 98/1/1 mg) in compared to the PEO group at the level of p = 0.009. ($$) a significant increase in the number of fibroblast cells in recipient (PEO/CU/Peptide; 98/1.5/0.5 mg) and (PEO/CU/Peptide; 98/1/1 mg) compared to the Cu group at the level of p = 0.002. Columns B: (*) a significant increase in the average volume density of the epidermis of the skin in recipient (PEO/CU/Peptide; 98/1.5/0.5 mg) and (PEO/CU/Peptide; 98/1/1 mg) in compared to the control group, respectively, at the level of p = 0.043 & p = 0.005. (†) a significant increase in the average volume density of the skin epidermis in recipient (PEO/CU/Peptide; 98/1.5/0.5 mg) and (PEO/CU/Peptide; 98/1/1 mg) in compared to group (PEO/CU; 98/2 mg), respectively, at the level of p = 0.011 & p = 0.013. Columns C & D: The results show no significant difference between the investigated groups.

Group	A; Fibroblasts Numerical Density ( × 10 ^3^per mm^3^) (Mean ± SD)	B; Volume Densities of the Collagen Bundles (Mean ± SD)	C; Volume Densities of the Vessel's (Mean ± SD)	D; Length Density (mm/mm^3^) (Mean ± SD)
Control	336.36 ± 72.70	0.701 ± 0.23	0.033 ± 0.006	20.86 ± 5.01
PEO/CU (98/2 mg)	350.37 ± 130.72	0.653 ± 0.138	0.029 ± 0.005	22.81 ± 3.14
PEO (100 mg)	313.98 ± 129.84	0.715 ± 0.11	0.035 ± 0.007	26.64 ± 6.45
PEO/CU/Peptide (98/1.5/0.5 mg)	510.87 ± 58.94 **^††$$^	0.867 ± 0.05*^†^	0.038 ± 0.008	23.86 ± 3.86
PEO/CU/Peptide (98/1/1 mg)	511.35 ± 72.15 **^††$$^	0.862 ± 0.06*^†^	0.031 ± 0.006	23.06 ± 8.06

## **4** **Discussion**

As nanotechnology and electrospinning have developed rapidly in recent years, ultrafine fibers in electrospinning have allowed for the uniform placement of additives and additional compounds on the surface of the polymer matrix. This leads to an increase in the desired performance of the polymer and its components. Additionally, electrospun polymer fibers provide strength and create more exposed surface area in a smaller volume, resulting in an increased use of these compounds in various industries such as food, industrial, pharmaceutical, dental, and medical equipment [[27], [28], [29], [30], [31], [32]]. For example, the use of electrospun nanofibrous membranes in tissue engineering, wound dressing, cancer diagnosis and treatment, medical protective equipment, and other fields has received much attention in recent years. The most promising nanocomposites being developed today are polymer-based nanocomposites. There are various polymers that can be used to fabricate nanocomposites, including polyethylene oxide, polyvinyl alcohol, polyglycolic acid, cellulose acetate, chitosan, collagen, polybutylene succinate, and alginate [[33], [34], [35], [36], [37]]. These polymers have various medical applications, such as manufacturing contact lenses, eye drops, embolization particles, artificial cartilage, bone damage repair, scars and wounds healing, and cartilage repair [[38], [39], [40], [41], [42], [43], [44], [45]]. Moreover, one of the most attractive polymers is polyethylene oxide. For instance, Chitosan/Polyethylene Oxide has orthopedic applications due to its osteoconductive effects [46]. Polyethylene oxide is a hydrophilic and nonionic linear polymer that is used in drug delivery and controlled drug release [47]. According to studies, the production of nanofibers with better properties can be achieved through electrospinning using polyethylene oxide polymer. This allows for better distribution of different additives and compounds on the surface of the fibers. The use of polyethylene oxide in the preparation of nanocomposites can be highly effective in soft tissue regeneration for tissue engineering [48,49]. Nowadays, the production of nanocomposites based on polymers with antibacterial properties, carrying various antibacterial nanoparticles or antimicrobial peptides such as silver, copper oxide, and defensin peptides, is of great importance due to the spread of antibiotic resistance among infectious agents [[50], [51], [52], [53]]. Copper nanoparticles, in particular, are of great importance due to their low cost and biomedical potential, including antibacterial, antioxidant, anti-inflammatory, anti-cancer, and neuroprotective activities [54,55]. Additionally, the use of natural peptide compounds such as plant defensins with antibacterial effects in nanocomposite structures is highly regarded. Furthermore, wound dressings are effective and valuable products that can enhance the regeneration of damaged skin. In these products, bio-absorption and bio-compatibility play essential roles, and defensin is one of the most promising materials for wound closure in the industry [[56], [57], [58]]. According to these documents, the aim of this study was to design a new electrospun polyethylene oxide polymer coated with copper nanoparticles and defensin. Additionally, our goal was to design new nanocomposites that are highly efficient and cost-effective for wound healing and to eliminate pathogenic microorganisms in the medical, pharmaceutical, textile, and food industries. Hence, we selected polyethylene oxide as the base structure due to its excellent properties, availability, affordability, and synthesized PEO/CuNPs/defensin nanocomposites using an electrospinning method with different ratios of copper/peptide (2/0, 1.5/0.5, and 1/1%) in the fixed polymer (98%). Then, the wound healing, tensile analysis, antibacterial, and antioxidant properties were investigated. Our analysis of the SEM image revealed that the PEO nanofibers were correctly synthesized and observed in a linear and parallel form without breaks. Moreover, the SEM images confirmed that the NPs did not aggregate into larger particles and were coated on the surface of PEO nanofibers with a regular distribution. In addition, our study revealed that the size of nanoparticles affects antimicrobial properties. In our study, the particle sizes of PEO, Cu NPs, and defensin were around 30–99, 90, and 15–20 respectively, which revealed effective antimicrobial properties. Furthermore, in our study, the stress-strain diagrams illustrate that the tensile strength of the designed fibers increased after the addition of NPs. The highest tensile strength is observed in the PEO/CuNPs/defensin (98/1.5/0.5 mg) nanocomposite, and the PEO/CuNPs/defensin (98/1/1 mg) nanocomposite shows the highest elasticity in comparison to PEO nanofibers alone. According to our results, the high tensile strength of PEO/CuNPs/defensin (98/1/1 mg) guarantees the biocompatibility and long-term performance of our wound dressing nanocomposites. It also verifies the effectiveness of the coatings and the resistance of the nanomaterial in the nanocomposite structures against harsh conditions such as physical conditions, mechanical stresses, or corrosion. Hence, the high tensile strength of the nanocomposite indirectly affects the wound healing process. Moreover, the PEO/CuNPs/defensin (98/1.5/0.5 mg) nanocomposite and PEO nanofiber illustrate the highest and lowest heat resistance, respectively. Hence, the thermal resistance of our designed nanocomposite assures its stability and durability in various applications, including those involving exposure to body heat. It also provides resistance to oxidation and particle degradation at very high temperatures. Consequently, it can be stated that the thermal stability and tensile strength of the polymer increase by adding NPs and plant peptides. This may be attributed to the different effects of nanoparticles and peptides. Furthermore, in this study, the designed nanocomposite showed antioxidant behavior. Additionally, when the wound is exposed to a polluted environment, the oxidation process can take place, causing a delay in wound healing. In fact, the antioxidant material can inhibit free reactive oxygen radicals (ROS) that are destructive to cells, damaged tissues, and body organs. Hence, the presence of antioxidant material in wound care products has led to inhibiting ROS-related tissue damage and reducing tissue inflammation at the wound site. Also, antioxidants make the composition more stable and prevent complications such as infections and scars. In our designed nanocomposite, defensin, as a natural agent, plays an important role in antioxidant properties. Besides, considering that wounds and even wound dressings have the possibility of being contaminated with environmental microbial agents, we decided to investigate the antimicrobial properties of the designed nanocomposite. We used the *S. aureus* standard strain as a representative of Gram-positive bacteria and the *E. coli* standard strain as a representative of Gram-negative bacteria. Our results showed the antimicrobial effects of the designed nanocomposite against both Gram-positive and Gram-negative bacteria. In fact, the presence of both CuNPs and defensin additives on the polymer surface reduced the growth rate of bacteria. According to factual evidence, defensins and copper nanoparticles have a range of molecular mechanisms against gram-positive and gram-negative bacteria. Defensins cause bacterial cell lysis by disrupting the integrity of the cell membrane or transport through bacterial cell walls, and they also disrupt bacterial metabolism. On the other hand, CuNPs show antibacterial effects through DNA degradation and membrane lipid peroxidation [57,58]. Furthermore, the PEO/CuNPs/defensin nanocomposite (98/1.5/0.5 mg), which shows the best results in the mentioned tests, exhibits an available area and porosity equal to 198.5983 m2/g. Similar to our research, several studies have been conducted on the design of nanocomposites with antibacterial components for wound dressing. For example, Kohsari et al. synthesized chitosan-polyethylene oxide nanofibrous material containing 0.25% and 0.50% Ag NPs with antimicrobial activity against both *S. aureus* and *E. coli* through the electrospinning technique for biomedical applications and wound dressing [59]. In another study, it was documented that designing chitosan/zinc oxide (CS/ZnO) nanocomposites and incorporating them into cotton fabrics can show antibacterial activity against *S. aureus* skin infection*, B. subtilis*, and *E. coli* [60]. Furthermore, diverse studies have documented that curcumin-loaded electrospun polycaprolactone/montmorillonite nanocomposites, polycaprolactone membranes with silver NPs, cellulose acetate nanofibers with bioactive glass NPs, graphene-reinforced electrospun chitosan/gelatin nanofibrous nanocomposite scaffolds, silver sulfadiazine-loaded electrospun ethyl cellulose/polylactic acid/collagen nanofibrous mats, and eugenol micro-emulsion reinforced with silver nanocomposite electrospun mats demonstrate antimicrobial activity and wound dressing application [[61], [62], [63], [64], [65], [66], [67], [68], [69], [70]]. As a result, electrospun nanofibers have been considered a relatively ideal material system for the design and manufacture of wound dressings in the past few decades. According to our results, the designed nanocomposite can be introduced as a new candidate with antibacterial and antioxidant effects for wound healing. We suggest that, due to the high potential of polyethylene oxide in the formation of nanofibers through electrospinning, it could be used as a scaffold to store compounds with natural origins and different nanoparticles with antibacterial properties to design new nanocomposites. Additionally, this nanocomposite is a suitable candidate in the pharmaceutical, medical, and therapeutic industries, as well as in the design of new wound healing dressings.

## 5 Conclusion

Today, wounds and their side effects have become problematic issues in medical science research. Dressings based on electrospun nanofibers show an effective role in wound healing due to their ability to maintain sterility, appropriate wound moisture, tissue compatibility, and antimicrobial effects. On the other hand, copper and defensin compounds have been experimentally used in studies as antimicrobial substances for years. In this study, PEO/CuNPs/defensin nanocomposites were successfully synthesized via the electrospinning method, and their wound healing, antibacterial, and antioxidant properties were investigated. According to the obtained results, the PEO/CuNPs/defensin (98/1.5/0.5 mg) nanocomposite is the best sample in terms of polymer and antibacterial properties. Therefore, it can be stated that the presence of CuNPs and defensin on the polymer surface improves antibacterial performance.

## Ethics approval and funding

This study was approved by the Department of Chemical Engineering, Shiraz Branch, 10.13039/501100002660Islamic Azad University, Shiraz, Iran, under grant number 16340201972002 and the Ethics approval Code 162486581.

## Data availability statement

Data included in article/supp. Material/referenced in article. No additional information is available for this paper.

## CRediT authorship contribution statement

**Sahba Eslaminezhad:** Validation, Resources, Project administration, Methodology, Investigation, Formal analysis, Data curation, Conceptualization. **Farhad Moradi:** Writing – review & editing, Writing – original draft, Validation, Supervision, Resources, Investigation, Data curation, Conceptualization. **Mahmoud Reza Hojjati:** Writing – original draft, Validation, Supervision, Resources, Methodology, Formal analysis, Conceptualization.

## Declaration of competing interest

The authors declare that they have no known competing financial interests or personal relationships that could have appeared to influence the work reported in this paper.
